# Making the invisible visible: a case study of the healing hearts initiative within the SEED wellbeing program in Australian healthcare

**DOI:** 10.3389/fpsyg.2026.1748690

**Published:** 2026-05-29

**Authors:** Padmini Pai, Andrea Knezevic, Danielle McCreadie, Jason Tanaka, Anoushka Andrade, Kylie Wright

**Affiliations:** 1 Illawarra Shoalhaven Local Health District, Warrawong, NSW, Australia; 2 School of Social Sciences, University of Wollongong, Wollongong, NSW, Australia

**Keywords:** healthcare staff, kindness, organizational culture, tangible tools, workplace wellbeing

## Abstract

**Background:**

Healthcare staff are experiencing increasing levels of burnout, moral strain, and disconnection. Organizational wellbeing strategies have often focused on individual resilience, with less attention to collective, relational or team-based approaches that nurture connection.

**Concept:**

The SEED Program, a workplace wellbeing intervention in healthcare, uses a strengths-based, staff-led approach to implementing wellbeing initiatives during work time. These initiatives are co-designed with staff and are intended to cultivate connection, creativity and kindness within the healthcare workforce. One of these initiatives is the Healing Hearts, which involves creating and sharing handmade felt hearts as tangible gifts of recognition and care. Conceptualized as a participatory kindness practice, this initiative illustrates how simple symbolic acts can be embedded into workplace culture to encourage connection, belonging and compassion.

**Context:**

Situated within a large regional health service in New South Wales, Australia, this case study examines the SEED Program and considers how initiatives such as Healing Hearts may complement existing organizational wellbeing frameworks and help address systemic risks of burnout.

**Approach:**

This paper examines the potential of wellbeing initiatives to embed routine, visible acts of kindness within healthcare environments through role-modeling, inclusive participation, and leadership endorsement. It positions this approach as a scalable, service-wide practice that aligns with broader organizational wellbeing strategies.

**Reflections:**

Early reflections indicate strong emotional resonance, multidisciplinary engagement, and the critical role of visible leadership support in enabling the uptake of wellbeing initiatives. These insights suggest that symbolic, yet relationally meaningful practices have the potential to strengthen workplace cultures of care and connection when thoughtfully integrated at scale.

## Introduction

Workplace wellbeing plays a critical role in shaping job performance, organizational culture, and overall employee engagement within healthcare, a sector facing persistent challenges of burnout, stress, and workforce shortages (De Neve et al., 2023; Gelencser et al., 2023). The financial benefits of wellbeing have been well documented, with reports indicating a $2.30 return for every $1 spent on wellbeing interventions in Australian organizations (Waddell et al., 2023). Beyond financial outcomes, investment in wellbeing correlates with increased employee commitment, stronger interpersonal relationships, and higher engagement, all contributing positively to workplace culture (Gelencser et al., 2023; Shifana and Sathyamoorthi, 2025). Consequently, workplace wellbeing is increasingly prioritized as organizations confront the urgent challenge of workforce retention amidst rising levels of stress and burnout (Gelencser et al., 2023).

This issue is especially pronounced in the healthcare sector, where high levels of staff burnout and turnover have far-reaching consequences for patient experience, the quality of care delivered, and the operational stability of healthcare services (Rotenstein et al., 2022). Healthcare staff are particularly vulnerable to burnout due to demanding workloads, the emotional toll of care provision, and the expectation of consistently high standards with minimal error (Lopez-Leon et al., 2021; Sexton et al., 2022). The effects of these pressures were dramatically intensified by the COVID-19 pandemic, with emerging data indicating that nearly one-third of healthcare staff are at risk of burnout as they navigate high-pressure environments, rapid change, and personal safety concerns (Wong et al., 2021). The cumulative stress has contributed to a critical shortfall in the global healthcare workforce, with forecasts projecting a gap of 15 million workers by 2030 (Lawrence et al., 2022).

Before the COVID-19 pandemic, healthcare organizations primarily invested in interventions aimed at improving patient safety, often allocating limited resources specifically for staff wellbeing (Lu et al., 2022). However, emerging evidence demonstrating the direct connection between staff wellbeing on patient safety has driven increased investment in wellbeing initiatives within the healthcare workforce (Lawrence et al., 2022). This shift accompanies a broader recognition of diverse wellbeing interventions available to healthcare staff, moving away from placing the sole responsibility of wellbeing on individual clinicians toward cultivating positive workplace cultures (Davids et al., 2024). The evolving integrated approach emphasizes social connectedness, interpersonal relationships, and a human-centered perspective to workplace wellbeing (Gupta et al., 2025; Waddell et al., 2023).

Globally, healthcare organizations have implemented diverse wellbeing initiatives to address workforce challenges such as burnout, stress and retention. These include resilience training, mindfulness and meditation programs, peer support networks, narrative medicine approaches, and structured team debriefings designed to promote psychological safety and mutual support (Kriakous et al., 2021; Lown and Manning, 2010). Notable international initiatives such as the Schwartz Center Rounds provide forums for healthcare staff to explore the emotional and social aspects of care, fostering empathy and team cohesion (Goodrich, 2012). Mindfulness-based stress reduction programs have demonstrated benefits in reducing anxiety and enhancing coping skills among healthcare workers (Kabat-Zinn and University of Massachusetts Medical Center/Worcester. Stress Reduction Clinic, 2005).

Research has indicated the buffering role of social supports and connectedness to protect against burnout within healthcare organizations, reflecting this human-centered approach to staff wellbeing (Yang and Khan, 2025). Environments that promote collegial support and mutual respect lead to stronger morale, a sense of belonging, and significantly lower rates of emotional exhaustion (Gilligan et al., 2019; Ward et al., 2022; Soderberg and Romney, 2022). Conversely, cultures marked by disconnection and distrust can hinder performance, impair communication, and erode team cohesion, ultimately compromising both staff and patient safety (Riskin et al., 2015). Despite evidence of the critical role of workplace environment on wellbeing outcomes, most interventions reported in the literature still focus on the individual, emphasizing a clear need for diverse strategies that promote positive work environments and relational connections (Karkou et al., 2024; Schwartz et al., 2020).

Individualized wellbeing interventions often frame burnout as a personal deficit rather than a relational or systemic issue, inadvertently placing responsibility on staff to cope (Davids et al., 2024). Such approaches have limited impact on psychological safety or collective morale and may unintentionally reinforce feelings of inadequacy or isolation (Gupta et al., 2025; Alcaraz-Córdoba et al., 2024). Without relational and organizational strategies, wellbeing efforts risk becoming fragmented, inequitable, or ineffective in environments characterized by high emotional labor and structural pressure (Knezevic et al., 2025).

This article aims to address this gap by highlighting an innovative wellbeing initiative that fosters a positive work environment within healthcare through encouragement of social connection and kindness among staff. By exploring relational, creative approaches to staff wellbeing, such initiatives have the potential to enhance emotional resilience, improve team cohesion, and ultimately contribute to improved patient care and workforce sustainability.

## The SEED program

Supporting the wellbeing of healthcare staff is increasingly recognized as essential for sustaining safe and effective healthcare delivery (Quirk et al., 2018), particularly in environments marked by workforce shortages, high pressure, and cumulative crises. The SEED Program is the workplace wellbeing intervention within which this case study is situated. Developed in the Illawarra Shoalhaven Local Health District (ISLHD), a large regional health service in New South Wales, SEED was established during a period of significant organizational strain as a staff-led, relational approach to workplace wellbeing (Mackay et al., 2021; Olcon et al., 2022; Pai et al., 2022). Rather than relying on traditional, individually focused initiatives, SEED emphasizes connection, collective care, and meaningful participation (Olcon et al., 2022; Knezevic et al., 2025). The following sections provide brief context on the environment in which SEED emerged and the principles that underpin it.

### Geographic and organizational context

The ISLHD serves over 400,000 people across a diverse coastal and regional area in New South Wales, Australia, supported by seven hospitals and multiple community health services. As one of the largest employers in the region, the district faces unique challenges in delivering scalable, inclusive wellbeing initiatives across varied geographic and demographic context.

### Origins and foundations of SEED

SEED originated in early 2020 following the Australian bushfires, emerging as a staff-led response to crisis. Rapid consultation with staff at a small rural hospital generated 84 practical suggestions that were distilled into a strengths-based framework—Stability, Encompassing, Endurance, and Direction (Mackay et al., 2021). SEED activities were implemented during work time and focused on psychological safety, connection, and collective efficacy (Olcon et al., 2022; Pai et al., 2022; Knezevic et al., 2025). Activities included peer-led discussions, creative workshops, and informal connection initiatives such as Coffee Buddies (Mackay et al., 2021). Rather than positioning staff as passive recipients of support, SEED explicitly recognizes healthcare workers as whole humans affected by systemic and environmental stressors, a perspective often absent from institutional wellbeing models. Developed amid bushfires, floods, and the COVID-19 pandemic, SEED fostered trust, peer support, and visible leadership endorsement.

Conceptually, SEED draws on three key orientations:

- *Strengths-based practice*, emphasizing existing capacities (Saleebey, 2006).
- *Relational leadership*, viewing wellbeing as co-constructed through social interaction (Uhl-Bien, 2006).
- *Kindness as an organizational value*, aligning with evidence linking compassion to reduced burnout (Scarlet et al., 2017).

SEED aligns with NSW Health priorities, including the Future Health policy and the Elevating the Human Experience strategy (NSW Health, 2022), and has spread across multiple ISLHD sites through local adaptation rather than formal mandate (Pai et al., 2022; Olcon et al., 2022).

### From SEED to the human experience (HEx) team

As SEED matured, its principles naturally extended into broader human-experience work. This evolution coincided with NSW Health's Elevating the Human Experience strategy, which emphasized relational care for patients, carers, staff, and volunteers (NSW Health, 2022). SEED's philosophy provided the foundation for establishing the Human Experience (HEx) team, which formalized and expanded these principles across the district. HEx did not replace SEED; rather, it institutionalized its values and broadened their application.

## Introducing healing hearts

Building on this foundation, the Healing Hearts initiative emerged from the SEED Program as a locally adapted version of the 1000 Hearts kindness movement (DeJonge, 2016). The initiative involves creating and sharing handmade felt hearts as tangible gifts of recognition and care. Originally conceptualized as a patient-focused compassion practice, ISLHD staff quickly recognized its potential as a staff-to-staff kindness initiative, particularly in the context of workforce fatigue and post-pandemic recovery. With support from NSW Health and guidance from the 1000 Hearts founder, HEx launched pilot activities in late 2024.

Initial rollout in Perioperative and Pediatric Units involved workshops, reflective conversations, and group heart-making sessions. Leaders modeled participation, and peer encouragement helped build momentum. Implementation was supported by improvement science methods and contextual feedback, enabling teams to adapt the practice to local needs. The HEx team coordinated workshops, engaged staff across multiple sites, and fostered leadership support to embed kindness practices into daily routines.

Healing Hearts now sits alongside other SEED- and HEx-supported initiatives such as Coffee Buddies, reflective conversations, creative workshops, and staff-led micro-initiatives. It was selected as the focus of this case study due to its symbolic resonance, accessibility across disciplines, and capacity to translate organizational values, particularly kindness and compassion, into tangible everyday practice.

### Healing hearts implementation

The insights presented here reflect preliminary, staff-reported perceptions of participation and implementation processes rather than formal outcome evaluation data. They are intended to illustrate contextual learning and practice-based considerations relevant to embedding kindness initiatives within healthcare organizations.

When hearts are shared with patients or staff, they are typically accompanied by a brief, unscripted message such as “This is a small token to let you know someone is thinking of you” or “This heart represents care and kindness from our team.” Staff are encouraged to adapt the language authentically rather than follow a standardized script. Leaders model this practice during workshops and ward-based activities, reinforcing that the gesture is not evaluative or performative-based, but a relational offering grounded in humanity. Staff and patient responses are largely described as appreciative and emotionally resonant, with some participants noting the unexpected impact of such a simple act.

### Healing hearts implementation timeline

Healing Hearts was implemented across ISLHD between August 2024 and November 2025 through a phased, incremental approach. The initiative began with a formally supported pilot and expanded across clinical and support services as local teams expressed interest and capacity. Table 1 summarizes the phases, sites, and delivery modes.

**Table 1:** Healing hearts implementation.

Phase	Period	Key activities	Sites/teams involved	Delivery mode
Pilot initiation	August–November 2024	Selection as NSW pilot site; planning and logistics; volunteer engagement; executive launch (“Gathering of Kindness”)	Perioperative unit (Wollongong Hospital); pediatrics unit	Facilitated workshops; leadership-led launch
Pilot implementation	January–March 2025	Heart-making sessions; reflective conversations; podcast recording; hearts used as reflective artifacts	Pediatrics unit	Facilitated staff sessions (day and night)
Early spread	March–May 2025	Heart pauses; cross-unit sessions; preparation for self-facilitated delivery; human experience week showcase	Transit Lounge; Shoalhaven Hospital; Physiotherapy Unit	Facilitated + self-facilitated
District expansion and evaluation	August–October 2025	Facilitator day; ripple-effect mapping; qualitative interviews; photovoice; program logic development	Multiple clinical and support services including drug and alcohol, intensive care unit and, the transitional aged care service	Mixed (facilitated, embedded practice)
Consolidation and celebration	November 2025	Gathering of kindness (drug and alcohol); workforce-specific engagement activities	District-wide	Celebration, reflection, workforce recognition

## Narrative summary of implementation

Healing Hearts commenced in August 2024 when ISLHD was selected as one of three NSW pilot sites, with coordination led by the Human Experience team. Following an executive-supported launch in November 2024, initial implementation focused on the Perioperative and Pediatrics Units. Early activities centered on facilitated heart-making sessions, reflective conversations, and leadership role-modeling to establish psychological safety and shared purpose.

Between March and May 2025, the initiative expanded beyond the pilot wards to additional clinical areas, including the Transit Lounge, Shoalhaven Hospital, and the Physiotherapy Unit. During this period, several teams began transitioning to self-facilitated delivery supported by peer champions and local leaders. Healing Hearts was also showcased during Human Experience Week, signaling organizational endorsement and readiness for broader adoption.

From August 2025, the initiative entered a district-wide expansion and evaluation phase. Structured qualitative methods, including ripple-effect mapping, photovoice, group interviews, and in-depth narrative interviews, were used to capture staff experiences and perceived impacts. A program logic model was developed to articulate the initiative's assumptions, mechanisms, and intended outcomes within the broader SEED and HEx frameworks. Low and medium-risk ethics approvals were obtained to support systematic inquiry.

Across the implementation period, Healing Hearts generated substantial relational engagement, including more than 500 recorded conversations, 11 in-depth interviews, one detailed case study, and multiple photovoice submissions. The initiative expanded beyond pilot wards to include the medical workforce and Nursing Unit Managers and fostered external collaboration with Sydney Local Health District and University of Wollongong students through shared education and reflection activities. These engagement metrics are presented as indicators of reach and participation rather than measures of effectiveness.

## Emergent ripple effects of healing hearts

Preliminary qualitative insights drawn from ripple effect mapping activities, reflective artifacts (including handmade hearts and written reflections), and early photovoice data suggest that the Healing Hearts initiative generates impacts that extend beyond individual participation. Using a ripple effect methodology to trace how a single act of kindness travels across contexts and applying *reflexive thematic analysis* informed by Braun and Clarke's (2021) 6 phase approach, emergent patterns indicate interconnected ripples at organizational, team, staff, and patient levels.

### Organizational and service-level ripples

At the organizational level, ripples were evident when senior and operational leaders introduced Healing Hearts within their teams, modeling participation and legitimizing kindness as a leadership practice. In several instances, leaders personally crafted and gifted hearts to acknowledge staff contributions, which prompted teams to identify locally relevant applications for both staff wellbeing and patient care. These actions often produced immediate secondary impacts, with staff independently offering hearts to patients or families within hours of the initial activity. Such observations suggest that leadership engagement acted as a catalyst for rapid diffusion of relational practice.

### Team-level ripples

Team-level ripples emerged as staff adapted Healing Hearts to their specific service contexts. Some clinicians extended the practice beyond hospital settings, incorporating heart-giving into community and home-based visits for individuals experiencing social isolation. These adaptations developed organically rather than through directive instruction, indicating that the initiative supported staff agency, creativity, and contextual responsiveness.

### Staff-level ripples

Staff-level ripples reflected the emotional and reflective impact of both making and giving a heart. Across written reflections and creative artifacts, participants described renewed connection to purpose, personal meaning, and appreciation of the relational dimensions of their work. A recurring motif, expressed through poetry and narrative reflections, was that offering kindness simultaneously deepened awareness of one's own emotional capacity and vulnerability. These insights align with understandings kindness as a reciprocal, rather than unidirectional, relational practice.

### Patient- and family-level ripples

Patient- and family-level ripples were most visible in acute moments of distress. Staff described offering hearts during high-intensity clinical encounters, including after-hours presentations and critical care situations involving uncertainty or grief. In these moments, the heart served as a tangible symbol of compassion when words felt insufficient, providing comfort to families while reinforcing staff perceptions of human connection amidst technical and emotional strain. Participants consistently emphasized that offering a heart felt like an honor rather than a task, underscoring its relational rather than transactional nature.

### Cross-level pattern

Across these ripple levels, a consistent pattern emerged: small, symbolic acts initiated within a supportive organizational context generated effects that extended outward in unanticipated ways, shaping interactions, practices, and meanings across the system. These preliminary findings suggest that Healing Hearts operates less as a discrete intervention and more as a relational mechanism through which kindness becomes visible, transferable, and embedded within everyday care practices.

## Conceptual integration: why SEED, HEx, and healing hearts fit together

The strength of the Healing Hearts initiative lies not only in its simplicity, but in its strategic alignment with the foundational principles of SEED and the HEx team. Each heart that is crafted and shared reflects a strengths-based, inclusive philosophy in which anyone, regardless of role or status, can contribute. This act of creating and gifting hearts models relational practice in real time, building meaningful connections and demonstrating that kindness is a tangible component of workplace culture rather than an abstract ideal.

Together, SEED, HEx, and Healing Hearts function as an integrated relational system rather than a collection of discrete wellbeing activities. SEED establishes the foundational conditions, psychological safety, staff agency, and relational practice, that enable wellbeing initiatives to take root. HEx provides the organizational governance, leadership visibility, and strategic alignment needed to legitimize and sustain these relational approaches at scale. Within this structure, Healing Hearts operates as a tangible, everyday expression of these principles, translating organizational values into simple, repeatable acts of kindness. This interconnected system allows symbolic gestures to generate broader cultural effects, as each component reinforces the others through shared values, visible modeling, and distributed participation across teams and services.

The interdependence of SEED, HEx, and Healing Hearts is central to the initiative's effectiveness. Healing Hearts, in isolation, would risk being perceived as tokenistic or unsustainable without the relational groundwork established through SEED or the organizational legitimacy provided by HEx. Conversely, SEED and HEx require tangible, everyday practices to translate relational values into lived behavior; without such enactments, aspirations to embed wellbeing remain abstract and difficult to sustain. The success of Healing Hearts within ISLHD reflects the presence of enabling conditions such as leadership visibility, protected time, psychological safety, and a history of staff-led relational practice. This layered approach suggests that the model is transferable beyond healthcare, provided organizations first cultivate relational foundations and structural supports before introducing symbolic practices tailored to local context.

Integrating Healing Hearts within the broader SEED and HEx frameworks addresses common critiques of workplace wellbeing programs that focus narrowly on individual self-care while overlooking the broader relational and cultural dimensions. Together, these initiatives reposition wellbeing as a shared, systemic responsibility embedded in everyday work. Healing Hearts operationalises this shift by making kindness visible and participatory, weaving collective wellbeing into routine practice rather than treating it as an optional add-on.

While the initiative offers clear value, challenges remain. Risks include perceptions of tokenism, questions about sustainability in resource-constrained environments, and reliance on volunteers to maintain momentum. However, governance structures, leadership sponsorship, and alignment with organizational strategies help mitigate these concerns. Senior executive support signals that Healing Hearts is a core organizational commitment rather than a peripheral gesture.

Importantly, Healing Hearts is designed to complement, not replace, broader structural reforms addressing workforce pressures and systemic barriers to wellbeing. The success of the initiative demonstrates the enduring impact of relational, participatory approaches within complex systems. When symbolic acts of kindness are supported by robust organizational structures, they create the conditions for genuine and sustainable cultural change.

The evolution of SEED to HEx, and now to Healing Hearts, demonstrates how grassroots initiatives can catalyze meaningful transformation, beginning with crisis response and progressing toward long-term institutional strategy. Embedding these principles strengthens organizational commitment to human-centered care and enhances capacity for compassion, resilience, and connectedness across the health system. This trajectory offers a model for cultivating more human-centered healthcare workplaces more human-centered, with benefits for staff wellbeing, organizational culture, and ultimately patient care.

## Discussion

The integration of Healing Hearts within the existing SEED and HEx frameworks demonstrates how relational, strengths-based, and kindness-focused approaches can meaningfully enhance staff wellbeing in healthcare environments. Preliminary ripple-effect findings show that even small symbolic acts, when supported by organizational structures, can generate multi-level impacts across staff, teams, patients, and services. These patterns reinforce the central premise of SEED: the sustainable wellbeing arises not from isolated individual interventions but from cultures that value connection, psychological safety, and shared humanity.

SEED's emphasis on relational practice aligns with evidence that wellbeing flourishes when staff experience competence, connection, and control, known as the “Three Cs” (Davids et al., 2024). International research similarly highlights that staff thrive in environments characterized by mutual respect, collegial support, and visible leadership engagement, and that social connectedness buffers against burnout and emotional exhaustion (Gilligan et al., 2019; Yang and Khan, 2025; Ward et al., 2022). The ripple effects observed in this study, particularly the rapid diffusion of kindness practices following leadership participation, illustrate how relational environments can amplify positive behaviors and strengthen team cohesion.

The findings also reflect a broader shift in the wellbeing literature away from individualized self-care models toward systemic, human-centered approaches (Karkou et al., 2024; Schwartz et al., 2020). Contemporary scholarship argues that wellbeing initiatives must address structural and cultural determinants, including workload, leadership visibility, and organizational values (Gupta et al., 2025; Waddell et al., 2023). Healing Hearts operationalises this shift by embedding kindness into everyday routines, enabling staff to enact compassion in ways that ae accessible, meaningful, and contextually adaptable. The initiative's organic adaptations, such as extending heart-giving into community settings, further demonstrate staff agency and creativity when relational practices are legitimized and supported.

While relational and kindness-based initiatives offer clear value, they cannot substitute for broader structural reform. Effective wellbeing strategies must operate across individual, relational, and organizational levels, addressing both symbolic and systemic dimensions (Lu et al., 2022; Lawrence et al., 2022). The preliminary findings suggest that Healing Hearts functions as a relational mechanism that that complements, rather than replaces, efforts to address workforce pressures, resource constraints, and systemic contributors to burnout. When supported by governance structures, leadership sponsorship, and alignment with organizational strategy, such initiatives can reinforce positive workforce cultures and contribute to improved staff retention, engagement, and patient experience (Gelencser et al., 2023; Waddell et al., 2023; Shifana and Sathyamoorthi, 2025).

Importantly, the early success of Healing Hearts also points to opportunities for developing a broader suite of relational, low-cost wellbeing practices. Complementary initiatives, such as fostering gratitude, sharing moments of awe or humor, or creating space for meaning-making, could be introduced periodically to maintain novelty and prevent any single practice from becoming routine or losing emotional resonance. Units might, for example, adopt two or more micro-initiatives per year, each grounded in co-design and adapted to local context. Such an approach would sustain engagement while reinforcing that relational care is an ongoing, collective endeavor rather than a one-off activity.

The evolution from SEED to HEx, and now to Healing Hearts, illustrating how grassroots, staff-led initiatives can catalyze meaningful cultural transformation. What began as a crisis-driven response has matured into a sustained organizational commitment to human-centered care. This trajectory demonstrates that relational practices, when embedded within supportive systems, can strengthen compassion, resilience, and connectedness across all levels of the health service. As such, the SEED-HEx-Healing Hearts continuum offers a compelling model for cultivating more humane healthcare workplaces, with benefits that extend to staff wellbeing, organizational culture, and ultimately patient care.

## Acknowledgment of constraints

While Healing Hearts is positioned as a core expression of organizational values rather than a peripheral activity, several conceptual and methodological constraints must be acknowledged. Conceptually, this initiative represents a single-site case study within one regional health service and does not include a comparison or control group. As such, the findings cannot be generalized or used to infer causality. Moreover, initiatives of this nature should not be viewed as substitutes for systemic structural reform; rather, they are complementary practices that sit alongside broader efforts to address workload pressures, staffing shortages, and systemic barriers to well being.

Methodologically, the initiative involved a relatively small sample of facilitators and required sustained engagement of staff working in high-demand environments. This raises questions regarding scalability and long-term sustainability, particularly in settings with limited resources. ISLHD secured dedicated funding for materials and allocated on-shift time for participation, conditions that may not be easily replicated elsewhere. Staff engagement itself functioned as a key resource, underscoring the dependence of such initiatives on local capacity and organizational support. There is also a risk that kindness-based practices may be perceived as tokenistic if not meaningfully integrated into organizational culture and reinforced by leadership.

To mitigate these challenges, a layered approach combining symbolic acts of kindness with structural support was adopted. This reflects lessons from SEED, which demonstrated that relational approaches could have enduring impact even within constrained systems. Governance structures and strong leadership sponsorship, including endorsement from the Chief Experience Officer at Ministry of Health, the ISLHD Chief Executive, the Director of Clinical Governance, and local Nurse Unit Managers, signaled organizational commitment and helped embed the initiative into routine practice. The structured pilot process, use of SEED networks, and direct engagement with the founder of the 1000 Hearts project further supported implementation.

The potential for Healing Hearts to be perceived as gendered was also considered. Although formal demographic participation data were not collected, workshop observations indicated engagement across genders and professional groups, particularly when the initiative was framed as collegial recognition rather than craft-based activity. Addressing gendered assumptions explicitly was important in normalizing participation and reinforcing that kindness is a professional competency relevant to all healthcare workers.

In summary, while the initiative lacks robust quantitative evaluation, its thoughtful design, strong conceptual grounding, and alignment with broader human-centered strategies provide a solid foundation for future research and implementation. Healing Hearts exemplifies how grassroots kindness initiatives, when supported by organizational structures, can contribute to cultural shifts toward compassion, resilience, and wellbeing within healthcare settings.

## Conclusion

The Healing Hearts initiative demonstrates how simple, tangible acts of kindness can strengthen connection, compassion, and belonging within healthcare workplaces. By embedding handmade felt hearts into everyday routines, the initiative made kindness visible and shared, affirming the emotional needs of staff alongside the technical demands of care.

Integrated within the SEED and HEx frameworks, Healing Hearts showed how grassroots, staff-led practices can complement organizational strategies and catalyze cultural change. Early traction, supported by executive endorsement and local leadership, highlighted that low-cost, relational gestures can boost morale and foster connection across clinical and non-clinical teams when grounded in authentic practice.

These insights reinforce the need to reframe wellbeing as a collective endeavor rather than an individual responsibility. Kindness-based initiatives, when supported by policy, leadership, and ongoing evaluation, can function as essential infrastructure for humanizing healthcare environments.

Looking ahead, robust outcome evaluation and economic analysis will be important to assess the long-impact and scalability. Cross-sector partnerships may further extend the reach of such approaches. Ultimately, the Healing Hearts journey underscores the value of creativity, connection, and sustained investment in human-centered strategies that uplift the healthcare workforce and, in turn, the people they care for.
